# Moving the needle on colorectal cancer genetics: it takes more than two to TANGO

**DOI:** 10.1038/s41416-018-0219-2

**Published:** 2018-10-04

**Authors:** Luis G. Carvajal-Carmona

**Affiliations:** 1 Genome Center, University of California, Davis, CA USA; 2 UC Davis Comprehensive Cancer Center, School of Medicine, University of California, Davis, CA USA; 3 Department of Biochemistry and Molecular Medicine, School of Medicine, University of California, Davis, CA USA

**Keywords:** Genetics research, Cancer genetics

## Summary

Colorectal cancer (CRC) is a leading cause of cancer incidence and mortality. It is well known that an important fraction of CRC risk is accounted by genetic factors. Identifying and characterising such factors may aid in identifying high-risk individuals who will benefit from effective preventive interventions.

## Preventative measures

Colorectal cancer (CRC) is a leading global cause of cancer incidence and mortality. After lung and breast cancer, it is the third most commonly diagnosed malignancy, with 1.36 million new patients diagnosed annually.^1^ Despite this large public health burden, most CRCs could be prevented through screening for pre-malignant lesions (polyps).^2^ Several CRC prevention programmes are already in place in countries with socialised medical systems, and these have resulted in a shift towards higher incidence of early-stage CRCs and a concomitant improvement in survival outcomes.^3–5^ Preventive interventions could benefit further from tools to identify high-risk groups. About a third of the CRC risk variance is attributable to inherited factors,^6^ and hence, genetic-based strategies offer great opportunities to identify high-risk individuals who could benefit from prevention and early detection.

The past decade has seen several advances in our understanding of the genetic aetiology of CRC. Through approaches that include linkage analysis, which involves genetic studies of disease co-segregation among affected relatives, and genome-wide association studies (GWAS), which involve comparing gene frequency data between CRC patients and healthy controls, 14 highly penetrant genes and >40 low-penetrance variants have so far been identified for CRC.^7,8^ These genes and variants only explain around 14% of the inherited risk of CRC^9^ and with most of these remaining relatively poorly understood. There is thus a clear need for additional discovery and functional studies to allow translation of these genetic findings into improved prevention strategies. In this issue of the *British Journal of Cancer*, four independent studies provide new data that furthers our understanding of CRC genetics. Two of these studies focus on novel gene discovery and the mechanistic understanding of highly penetrant CRC forms, whereas the other two papers focus on GWAS and post-GWAS approaches to study CRC aetiology and survival.

## New genes and new mechanisms

Evidence for a new risk variant on chromosome 1q32 was provided by Schubert et al.,^10^ building on previous studies that demonstrated the association of a nearby region (1q41) with the risk of CRC and multiple adenomas.^11,12^ The authors used two independent gene identification approaches (homozygosity mapping and linkage analysis) to identify a rare non-synonymous single-nucleotide variant (nsSNP, p.Asp1432Glu) in the *MIA3* gene (also known as *TANGO*, a gene not previously known to be associated with CRC). Co-segregation studies together with tumour gene and protein expression analyses provided supporting data for a role of *TANGO* p.Asp1432Glu in CRC tumourigenesis. Further independent studies are warranted to confirm the causal role of this *TANGO* variant in CRC.

The second study focussed on understanding the mechanisms by which epigenetic mutations lead to CRC in Lynch Syndrome (LS), the most common CRC-predisposing syndrome.^13^ Estela Dámaso and collaborators^14^ investigated the mechanism underlying constitutional primary *MLH1* epimutations. Individuals with these epimutations represent around 2% of all mutation-negative cases suspected of LS and typically have severe CRC manifestations.^15^ In this condition, *MLH1* epimutations typically arise de novo and lead to the soma-wide inactivation of the *MLH1* allele by methylation, which in turns leads to CRC. *MLH1*, a DNA mismatch repair gene, is also methylated in sporadic CRC where it explains most CRCs with microsatellite instability. Yet the manifestation is different between sporadic CRC and constitutional primary *MLH1* epimutation, with the sporadic manifestation exhibiting a colorectal tissue-specific alteration, rather than soma-wide. In their study, 12 constitutional primary *MLH1* epimutation carriers along with 61 LS patients and 41 controls were analysed with genome-wide methylation arrays to identify differentially methylated regions (DMRs) between epimutation and non-epimutation carriers. This analysis found that the only DMR was a CpG island encompassing the *MLH1* and *EPM2AIP1* genes, suggesting that constitutional primary *MLH1* epimutation-driven CRCs are different from sporadic *MLH1* methylated CRCs, where they exhibit a focal, rather than genome-wide, methylation pattern.

Analysing the heritability of *MLH1* epimutations, the authors revealed that the epimutations were neither inherited nor passed to descendants, because they were not detected in affected relatives despite harbouring the epimutation-bearing haplotype. This latter result is important, because it shows that constitutional primary *MLH1* epimutations experience inter-generation erasure. Such information is important for genetic counselling of *MLH1* epimutation carriers and for our understanding of CRC tumourigenesis in patients with this condition.

## Genetic aetiology, functional studies and studying the genetics of CRC survival

Most GWAS-identified variants map to non-coding regions, and they likely increase risk by regulating transcript levels of nearby genes.^8^ Identifying genes whose transcripts are under genetic control (called eGenes) is thus useful to discover new CRC variants and to understand the mechanisms by which GWAS alleles increase CRC risk. In order to link genetic variation with gene expression patterns, Moreno et al.^16^ discovered expression quantitative trait loci (eQTLs) in colon tissue from paired colon tumour/adjacent normal samples and from normal colonic biopsies obtained from healthy donors. The study identified 363 colonic eGenes, many of which overlapped with those in GTEx (a publicly available eQTL database) or with those identified in a previous study.^17^ Interestingly, of the 37,099 eQTLs discovered in this study, only 4,858 were identified both in normal and tumour tissue, raising the possibility that some of these might be good candidates for CRC tumourigenesis studies. These data are now publicly available through a dedicated website and they represent a great resource in post-GWAS studies of CRC. The final study was aimed at identifying nsSNPs affecting CRC survival.^18^ Despite having sufficient statistical power in this GWAS study to detect nsSNPs with modest effects on survival, the study failed to find genome-wide significant associations, suggesting that survival may be influenced by rarer nsSNPs or by common non-coding variants. Future studies, therefore, should not only increase sample size to enable the detection of milder effects but should also include additional types of genetic variation that may have effects on prognosis.

The studies published in this issue help move the needle towards an improved understanding of the role of genetic factors in CRC aetiology and survival. They show that multiple approaches are needed to identify and characterise CRC genes and suggest that future studies, particularly those focussing on complex phenotypes such as survival, will require collaborative efforts by the international research community.

## Disclaimer

The content is solely the responsibility of the author and does not necessarily represent the official views of the National Institutes of Health.
